# AuNPs@Al-TDC Metal–Organic Framework: A Hybrid Nanostructure for 3D-Printed Electrochemical Sensors Targeting Diuron

**DOI:** 10.1021/acsomega.5c11545

**Published:** 2026-02-11

**Authors:** Raylla Santos Oliveira, Hudson Batista da Silva, Esther de Jorge Duarte, Cassiano Cunha de Souza, Wallace Burger Veríssimo de Oliveira, Charlane Cimini Corrêa, Gustavo Fernandes Souza Andrade, Maria Auxiliadora Costa Matos, Thalles Pedrosa Lisboa, Renato Camargo Matos

**Affiliations:** † Departamento de Química, 28113 Universidade Federal de Juiz de Fora, Juiz de Fora, Minas Gerais 36026-900, Brazil; ‡ Faculdade de Ciências Exatas e Tecnologia, 186079 Universidade Federal da Grande Dourados, Dourados, Mato Grosso do Sul 79804-970, Brazil

## Abstract

The integration of sustainability principles, green chemistry, and circular economy concepts enables the recycling of polymers such as acrylonitrile-butadiene-styrene (ABS) into innovative and functional devices. In this context, advances in composite materials and conductive filaments are essential for the development of inexpensive and sustainable electrochemical sensors. This study presents a sensor (AuNPs@Al-TDC/3D-CPE) fabricated via 3D printing using recycled ABS filaments, modified with the Al-TDC metal–organic framework (MOF) and gold nanoparticles (AuNPs). The MOF composite was synthesized from tetrachloroauric acid, thiophene-2,5-dicarboxylic acid, dimethylformamide, and formic acid under microwave-assisted hydrothermal conditions, and in a postsynthesis modification, the AuNPs were incorporated. The hybrid materials underwent a comprehensive physicochemical evaluation through spectroscopic, imaging, and electrochemical approaches, confirming both the successful incorporation of the modifiers and the structural integrity of the sensing platform. For its analytical application, the pesticide Diuron was selected as the target molecule for the development of a differential pulse voltammetry (DPV) protocol, whose procedure was systematically optimized using the AuNPs@Al-TDC/3D-CPE. The sensor exhibited a well-defined linear dynamic range between 1.0 and 20.0 μmol L^–1^, achieving a detection limit of 0.040 μmol L^–1^. Precision was confirmed by relative standard deviations consistently below 7%. Accuracy was further validated through recovery assays in fortified water samples, yielding values between 97% and 105%. The proposed sensor is efficient, cost-effective, and environmentally friendly, representing a promising alternative for the monitoring of emerging contaminants in alignment with the sustainable development goals.

## Graphical abstract

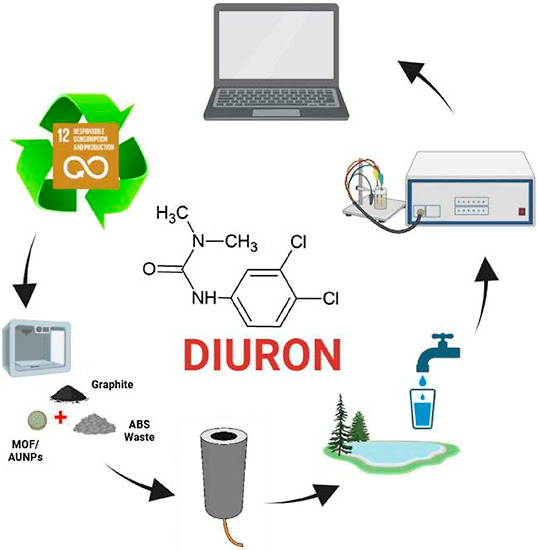

## 1 Introduction

Brazil, as a global agricultural powerhouse and one of the largest consumers of pesticides, has approximately 40% of its pesticide market composed of herbicides, particularly diuron (DIU).^1^ The herbicidal activity of DIU stems from its ability to disrupt the photosynthetic process in plants. Its action mechanism consists of inhibiting the plastoquinone binding site in photosystem II, preventing the transfer of electrons from photosystem II to plastoquinone. This disruption interrupts the photosynthetic electron transport chain and compromises the plant’s ability to transform light energy into chemical energy.^3^ Extensive sugar cane cultivation in Brazil relies heavily on DIU for effective weed control, making it a key component of the country’s agricultural practices.^2^ However, the use of DIU extends beyond agriculture, as it is also employed in antifouling paint formulations to inhibit the adhesion and proliferation of marine organisms on ship hulls and submerged surfaces, which constitutes an important nonagricultural source of environmental contamination.^3^ This dual-use scenario contributes to the widespread occurrence of DIU in aquatic environments and underscores the need for sensitive and reliable analytical methods for water quality monitoring.

Several analytical strategies have been described for the determination of DIU, covering high-performance liquid chromatography,^4^ capillary electrophoresis,^5^ gas chromatography,^6^ infrared spectroscopy,^7^ mass spectrometry,^8^ as well as electrochemical approaches.^9^ In recent years, the incorporation of chemical modifiers has played a crucial role in the development of electrochemical sensors with improved sensitivity, selectivity, and analytical performance, achieved through simple and rapid modifications of the sensor interface.^10^ Within this context, nanomaterials have gained prominence as electrode modifiers owing to their distinctive physicochemical features, including large surface area, superior electrical conductivity, and adaptable surface chemistry.^11^ More recently, the integration of electrochemical sensors with MOFs has emerged as an attractive approach, offering synergistic effects that significantly enhance electroanalytical performance.^12^

Metal–organic frameworks are a class of coordination polymers with porous dimensions constructed from metal ions or metal clusters interconnected by organic ligands. MOFs have highly ordered and porous three-dimensional networks with adjustable pore sizes and shapes, giving this type of material outstanding characteristics such as high surface area, thermal stability, and high crystallinity.^13^ These features have enabled their application across a broad spectrum of fields, including gas storage,^14^ molecular separations,^15^ catalysis,^16^ and the development of electrochemical sensors.^17^ In addition to increasing the surface area of sensors,^18^ MOFs can be tailored to enhance chemical selectivity, making them effective for the detection of a variety of target compounds.^19^ However, a primary limitation for their use in electrochemical sensors is their inherently poor electrical conductivity. To address this, doping MOFs with metallic nanoparticles has been widely employed to facilitate electron transfer processes.^20^ Another strategy involves dispersing MOFs within carbon-based materials to fabricate carbon paste electrodes, thereby improving conductivity and sensor performance.^21^

Metal nanoparticles exhibit unique physical and chemical properties compared to their macroscopic counterparts.^22^ When AuNPs are incorporated into the MOFs, hybrid AuNPs@MOF composites are formed. The high porosity and large surface area of MOFs facilitate the encapsulation of AuNPs within their cavities.^23^ These composite materials demonstrate remarkable performance in sensing applications due to the synergistic interactions between AuNPs and the MOF structure, which combine the high conductivity and surface plasmon activity of metals with the selectivity and structural stability of MOFs.^24^ Moreover, AuNPs@MOF composites have attracted significant attention in the development of modified electrodes for voltammetric techniques.^25^ The incorporation of metallic nanoparticles into a MOF matrix greatly enhances the sensitivity and selectivity of electrochemical responses, as the MOF acts as a porous and stable platform that promotes the diffusion of analyte species and facilitates electron transfer between the electrode surface and the analyte.^24^

3D printing, also known as additive manufacturing, has revolutionized numerous industries by enabling the creation of complex objects with exceptional precision and efficiency. Among the most commonly employed methods is fused deposition modeling (FDM), where a thermoplastic filament is fed into a heated nozzle, melted, and deposited layer by layer following a digital design.^26^ This versatile technology enables the production of intricate geometries using a broad range of materials, making it an ideal tool to support circular economy practices. The circular economy promotes the reduction of natural resource consumption and waste generation, minimizes environmental impacts, and facilitates the recovery of materials and energy.^27^ Polymeric materials modified with conductive additives represent a promising avenue for developing electroanalytical methods aimed at monitoring emerging contaminants. Materials such as polylactic acid (PLA) and recycled polyethylene terephthalate glycol (rPETg) have been demonstrated success in producing conductive filaments that exhibit excellent electrochemical performance alongside superior mechanical and thermal properties.^28,29^ Various modifications incorporating metal nanoparticles, metal oxides, and carbon-based derivatives have been extensively reported.^30^ However, there has been limited focus on functionalizing promising 3D-printing materials with MOFs, with most studies concentrating on applications such as heavy metal and glucose detection.^31,32^

In this study, we describe the development of a novel composite material based on the recycling of 3D printing waste from the acrylonitrile-butadiene-styrene polymer and uniquely modified with a MOF, composed of octahedral chains of AlO_4_(OH)_2_ connected by thiophene ligands, in combination with gold nanoparticles, designated as AuNPs@Al-TDC. The composite material was used to construct a 3D printed electrochemical sensor (AuNPs@Al-TDC/3D-CPE). This innovative platform was successfully applied for the sensitive determination of diuron in water samples from rivers, lakes, and taps.

## 2 Materials and Methods

### 2.1 Chemicals, Solutions, and Samples

Standards for DIU, carbendazim (CBZ), glyphosate (GLY), and 2,4-dichlorophenoxyacetic acid (2,4-*d*) were obtained from Sigma-Aldrich (St. Louis, USA). Solvents and reagents, including acetone, chloroform, boric acid, glacial acetic acid, phosphoric acid, sodium nitrate, sodium nitrite, calcium chloride, sodium carbonate, and potassium chloride, were obtained from Vetec Química Fina Ltd.a. Tetrachloroauric acid (HAuCl_4_), thiophene-2,5-dicarboxylic acid (TDC), dimethylformamide (DMF), and methanoic acid (formic acid, MET) were also acquired from Sigma-Aldrich. Aluminum chloride hexahydrate (AlCl_3_.6H_2_O) was supplied by Êxodo Científica (São Paulo, Brazil), and graphite powder was sourced from Synth (Diadema, Brazil). All reagents were employed as received, without any additional purification.

The DIU stock solution was prepared in ethanol, whereas the CBZ stock solution was prepared in 0.1 mol L^–1^ sulfuric acid. The Britton–Robinson (BR) buffer was prepared by combining equimolar (0.1 mol L^–1^) solutions of boric acid, glacial acetic acid, and phosphoric acid. The pH was adjusted between 2.0 and 12.0 using a Digimed DM-22 potentiometer (São Paulo, Brazil) by adding 1 mol L^–1^ solutions of sodium hydroxide or hydrochloric acid.

The determination of DIU using the AuNPs@Al-TDC/3D-CPE sensor by DPV was performed in tap, lake, and river water samples, collected from laboratory, Manacás Lake, and Paraibuna River, respectively, located at Juiz de Fora (MG, Brazil). All samples were diluted 10-fold with BR buffer in 5.00 mL volumetric flasks. Lake and river samples were filtered to remove particulate matter before analysis. The samples were then spiked at three concentration levels: 5.0, 10.0, and 15.0 μmol L^–1^.

### 2.2 Synthesis of the AuNPs@Al-TDC

The Al-TDC MOF was prepared following the method described in the work of Tannert et al.,^33^ where 0.24 mmol of AlCl_3_·6H_2_O and 0.18 mmol of TDC were solubilized in a mixture of water/DMF (4:1, v/v). The reaction was conducted under reflux at 135 °C for 24 h. The resulting product was washed with excess DMF to remove reagent residues. This process yielded a microcrystalline powder. The AuNPs were synthesized according to the method reported by Frens^34^ and subsequently incorporated into the Al-TDC structure. AuNPs was introduced into the MOF structure by mixing 500 μL of AuNPs with 500 μL of MOF suspension (2 mg mL^–1^). The mixture was stirred overnight, then centrifuged and left to dry at room temperature.

The Al-TDC and AuNPs@Al-TDC were characterized by X-ray powder diffraction (XRD) patterns were recorded on a Bruker D8 Advance Da Vinci diffractometer in Bragg–Brentano θ–θ geometry and using a Cu–Kα source (λ = 1.54056 Å), Ni filter, and LynxEye linear detector at 40 kV and 40 mA used an angular range of 5° to 70°. Besides, Fourier-transform infrared (FTIR) spectra were recorded on a Bruker Vertex 70 spectrometer equipped with a Hyperion 3000 microscope. Measurements were performed in attenuated total reflectance (ATR) mode with a diamond crystal. The spectra were collected with a resolution of 4 cm^–1^ over the spectral range of 400–4000 cm^–1^. The Raman spectra were obtained in a Bruker SENTERRA Raman spectrometer equipped with an Olympus BX51 optical microscope. The samples presented a high sensitivity to the laser beam. To mitigate this problem, Raman spectra were acquired by mapping 25 sample points using a 633 nm laser at 0.2 mW, with an accumulation time of 15 or 30 s, and a 20× objective lens (NA = 0.40). The 25 spectra were averaged to improve signal-to-noise ratio. Scanning electron microscopy (SEM) images of the samples were obtained using an FEI Quanta 250 instrument with an electron acceleration voltage of 30 kV. UV–vis–NIR absorption and diffuse reflectance measurements were obtained on an Ocean Optics USB 2000 + XR1-ES, NIR 256–2.1 spectrophotometer in the 200 to 2800 nm range.

### 2.3 Manufacturing of AuNPs@Al-TDC/3D-CPE Sensor

The working electrode was constructed by immobilizing a conductive paste on a 3D printed support using a GTMax3D CORE model A2 V2 3D printer (Americana, Brazil), equipped with a 0.4 mm hot-end nozzle, operating at an extrusion temperature of 230 °C and a heated bed at 100 °C, as shown in Scheme 1. The supports, modeled using AutoCAD software and sliced using Simplify software, were printed with 1.75 mm ABS filament (3D Tech Brasil Ltd. a, Joinville, Brazil) in a cylindrical shape (3 mm radius and 35 mm height) with 30% infill and a layer height of 0.1 mm. A concentric cylindrical opening with a radius of 1 mm was included for inserting a copper wire to be used to establish electrical contact. The conductive paste was immobilized in one of the ends, which contained a cylindrical cavity (2 mm radius and 3 mm depth).

**Scheme 1:**
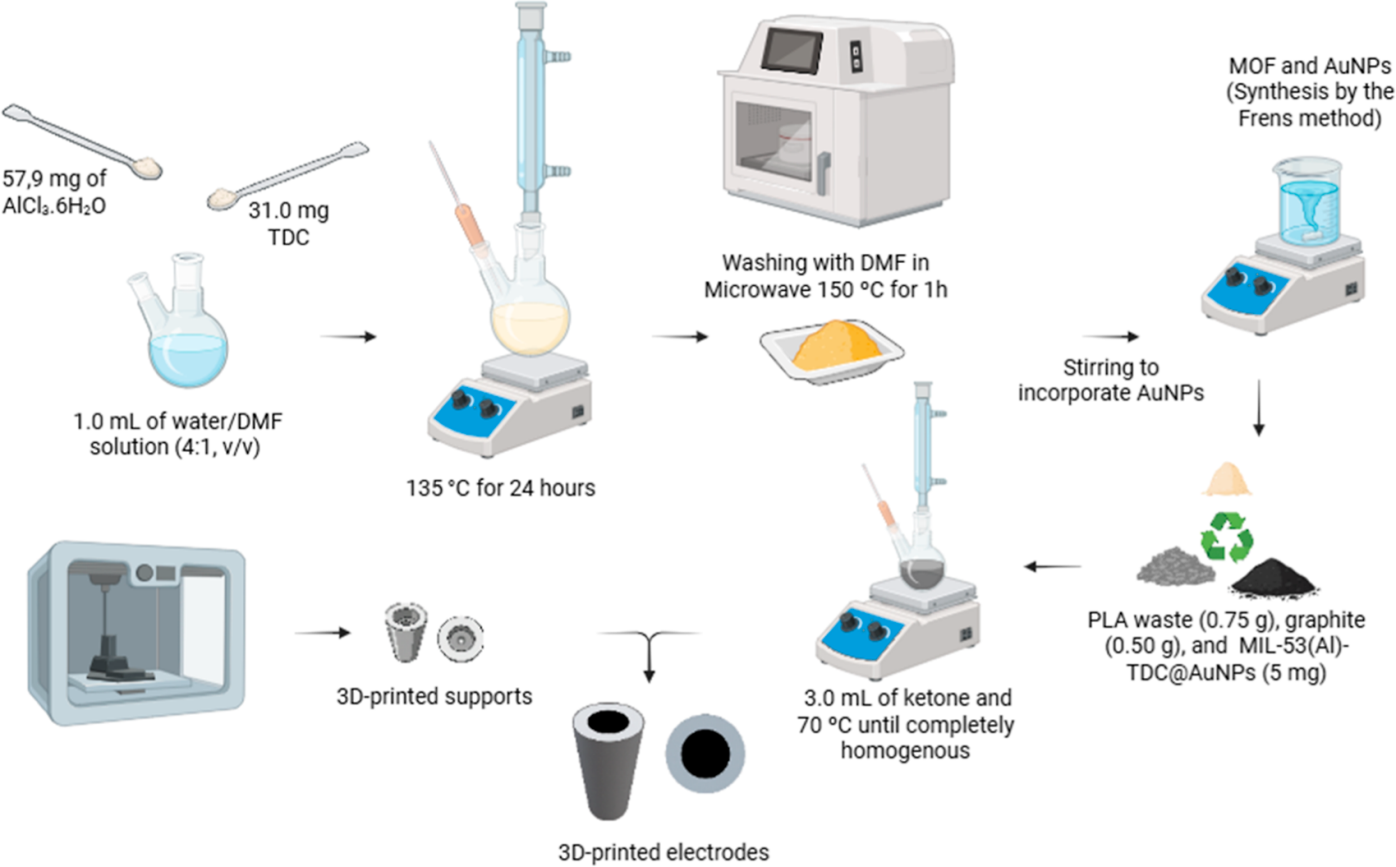
Al-TDC Synthesis, AuNPs Functionalization, 3D Printing of the Supports, and Fabrication of the AuNPs@Al-TDC/3D-CPE

The conductive paste was prepared by grinding the ABS waste. Then, 0.75 g of ABS was weighed and mixed with 3 mL of a mixture of acetone and chloroform (3:1 v/v). The mixture was then heated to 70 °C and kept under stirring and reflux until the polymer was completely dissolved. Subsequently, 0.5 g of graphite and 5 mg of AuNPs@Al-TDC were added, and the mixture was stirred for 30 min until a homogeneous conductive paste was obtained.

The 3D-printed supports were filled with the conductive paste, and the electrodes were dried in an oven at 50 °C for 2 h. After drying, the electrode surface was mechanically polished using water sandpaper of progressively finer grits (400, 1200, and 2000) to remove excess material and achieve a smooth, uniform finish, as shown in Figure S1 of the Supporting Information. The morphology of the sensor surface was characterized using the scanning electron microscopy (SEM) images, obtained using a Hitachi TM3000 microscope (Tokyo, Japan) at 15 kV, and energy-dispersive X-ray spectroscopy (EDX) analyses were performed on a Hitachi TM3030 tabletop microscope equipped with SwiftED3000.

### 2.4 Electrochemical Measurements

Electrochemical measurements were carried out using a PGSTAT-128 N potentiostat/galvanostat (Metrohm, Herisau, Switzerland) controlled by NOVA software (version 2.1.6). The techniques employed included cyclic voltammetry (CV), differential pulse voltammetry, and electrochemical impedance spectroscopy (EIS). Experiments were conducted in a conventional three-electrode system within a 5 mL electrochemical cell, comprising the AuNPs@Al-TDC/3D-CPE as the working electrode, a platinum wire as the counter electrode, and an Ag|AgCl_(s)_, KCl_(sat.)_ as reference electrode.

EIS measurements were performed using 5 mmol L^–1^ potassium ferricyanide in 0.5 mol L^–1^ KCl as the redox probe. Data were collected at an applied potential of +0.2 V vs. Ag|AgCl_(s)_, KCl_(sat.)_ over a frequency range of 10 kHz to 0.1 Hz, using a sinusoidal perturbation of 10 mV and 10 points per decade.

The electrochemical behavior of DIU at the modified sensor was investigated via DPV and CV. The effect of electrolyte pH on DIU’s redox prob response was evaluated by DPV in 0.1 mol L^–1^ BR buffer over a pH range of 2.0 to 12.0. CV experiments were performed to study the mass transport mechanism of 100 μmol L^–1^ DIU by varying the scan rate from 50 to 250 mV s^–1^ in 0.1 mol L^–1^ BR buffer at pH 12.0.

Moreover, DPV parameters were optimized for DIU determination, examining pulse amplitude (10–100 mV) and step potential (1–8 mV). After optimization, an analytical calibration curve was constructed for DIU concentrations ranging from 0.99 to 19.2 μmol L^–1^. All DPV measurements were carried out in 5 mL of 0.1 mol L^–1^ BR buffer at pH 12.0 as the supporting electrolyte.

## 3 Results and Discussions

### 3.1 AuNPs@Al-TDC Characterization

The MOF Al-TDC has the molecular formula [Al­(μ–OH)­(TDC)] and is isoreticular with the organic ligand 1,4-benzenedicarboxylic acid. In this framework, Al^3+^ ions exhibit an octahedral coordination geometry and are coordinated by six oxygen atoms, four from distinct TDC ligands and two from bridging hydroxyl groups (μ–OH). The resulting structure features high structural flexibility, besides pores with an approximate diameter of 9.2 Å.^35,36^

Figure 1A shows the XRD patterns of both pristine and AuNPs@Al-TDC. The characteristic diffraction peaks of the MOF remain largely unchanged following the incorporation of AuNPs, indicating that the crystalline structure of Al-TDC is preserved. However, additional peaks at 38.1° (111), 44.3° (200), 64.5° (220), and 77.3° (311), which are characteristic of the crystal lattice pattern of metallic gold and attributable to AuNPs,^35^ confirming their successful incorporation into the porous structure of the MOF.^37^ Additionally, the Al-TDC peaks remain in approximately the same positions after the addition of the AuNPs, suggesting structural stability of the MOF, which corroborates the maintenance of porosity and increase in the electroactive area of the modified sensor.

**Figure 1:**
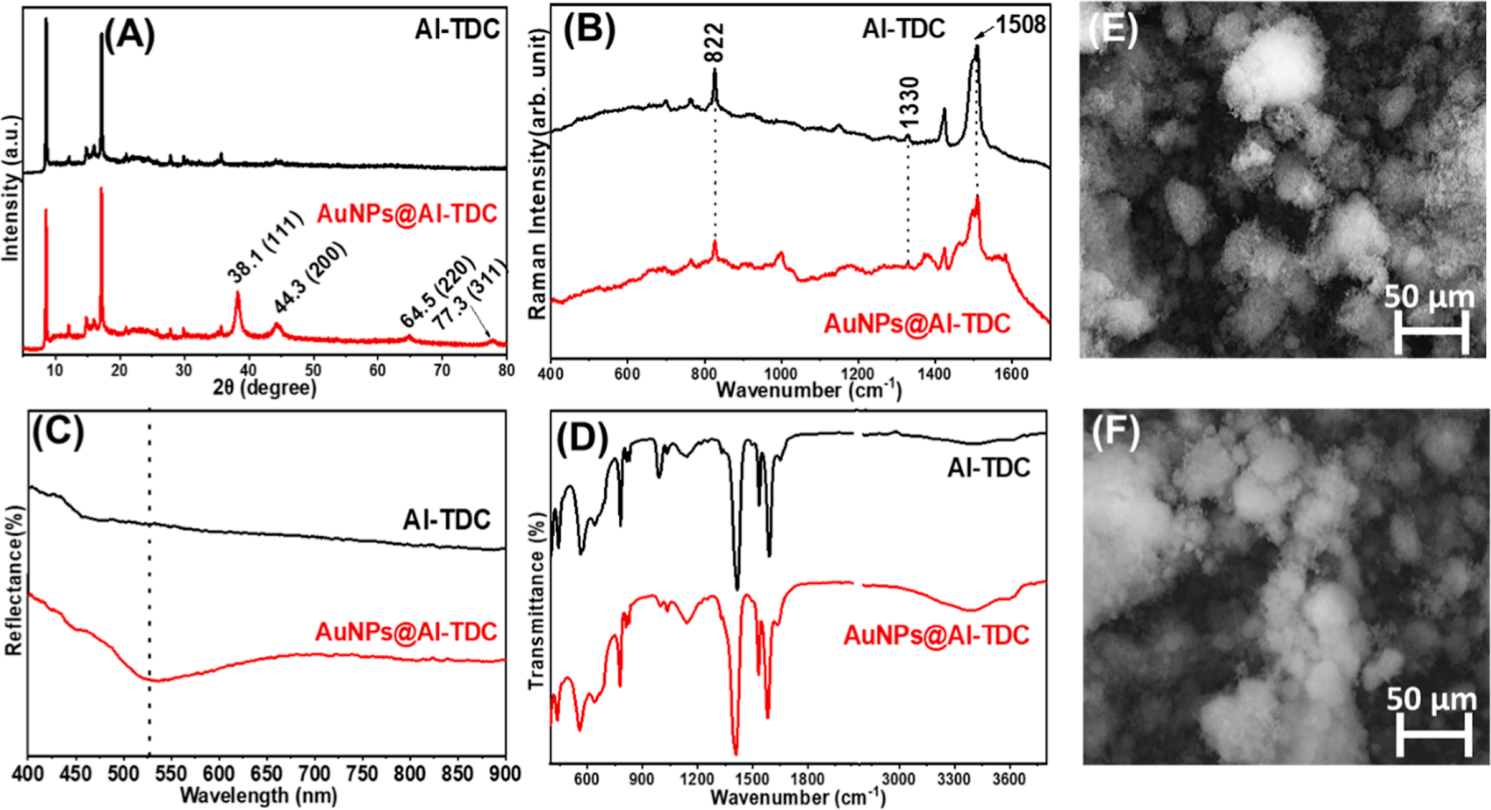
Structural and morphological characterization of pristine and AuNP-modified AuNPs@Al-TDC: (A) XRD patterns, (B) Raman spectra, (C) UV–Vis–NIR spectra, (D) FTIR spectra, and (E,F) SEM images of Al-TDC and AuNPs@Al-TDC, respectively. The results confirm AuNP incorporation without significant changes in the MOF structure or morphology.

Figure 1B shows the Raman spectra of the pure MOF and the AuNPs-modified MOF. Although slight variations can be observed, no significant changes occurred in the overall spectral profile after the incorporation of AuNPs into the MOF structure. The main bands were observed at 1508 and 1330 cm^–1^, corresponding to the antisymmetric and symmetric stretching vibrations of the carboxylate (COO^–^) groups, respectively, and at 822 cm^–1^, which is assigned to the C–H bending mode, possibly from modes linked to the aromatic structure of TDC. Figure 1C shows the UV–Vis–NIR diffuse reflectance spectra of the pure MOF and the gold-modified sample. Initially, relatively high reflectance is observed for Al-TDC over the 400 to 900 nm range. However, after the AuNPs modification, the reflectance decreases significantly with the appearance of a band at approximately 530 nm, indicating light absorption by the AuNPs, a surface plasmon effect characteristic of spherical nanoparticles indicating the successful incorporation of AuNPs into the MOF in the AuNPs@Al-TDC hybrid material.

Figure 1D displays the FTIR spectra of Al-TDC and AuNPs@Al-TDC. The spectral profiles of both materials are nearly identical, indicating that the chemical structure of the framework remains intact after gold nanoparticle modification. The bands at 1590 and 1415 cm^–1^ correspond to the asymmetric and symmetric stretching vibrations of the carboxylate groups (COO^–^), respectively, and are evident in both spectra. A band at 1530 cm^–1^, attributed to the C=C stretching of the aromatic ring, is also present in both samples. Furthermore, the band at 781 cm^–1^, assigned to the C–S stretching vibration, appears in both species, confirming the preservation of the thiophene moiety.^36,38^ Finally, Figure 1E,F exhibit the SEM images of the Al-TDC MOF and AuNPs@Al-TDC, respectively. The morphology of the particles observed is consistent with previously reported Al-TDC materials, which are known to form microcrystalline agglomerates with irregular shapes, rather than well-defined crystalline facets, as documented in the literature by Tannert et al.^33^ Furthermore, the morphology of the material remained unchanged after the AuNPs were incorporated into the MOF structure, which indicates that the incorporation of AuNPs maintained the morphology of the materials. Additionally, Figure S2 of the Supporting Information shows the maps of C, Al and Au from the SEM images, showing the distribution of species in the structure of the proposed MOFs and the 3D printed sensor.

### 3.2 Electrochemical and Morphological Characterization of the AuNPs@Al-TDC/3D-CPE Sensor

The electrochemical performance of the AuNPs@Al-TDC/3D-CPE sensor was evaluated by CV and EIS and compared to the unmodified 3D-CPE sensor using a 5 mmol L^–1^ potassium ferricyanide redox probe in a 0.5 mol L^–1^ potassium chloride solution, as shown in Table 1. The CV voltammograms (Figure 2A) reveal a well-defined profile characteristic of the reversible redox peaks of the potassium ferricyanide probe, evidenced by the ratio between the anodic and cathodic peak currents of 0.959 and 1.094 for the unmodified and modified electrodes, respectively. Furthermore, an increase of approximately 1.5 times in the peak currents was observed, which indicates a better electronic transfer capacity of the modified electrode compared to the unmodified one. The porous structure of MOF is known to facilitate the transport of ions and molecules, and the presence of AuNPs associated with the electroactive sites of the proposed electrode contributes to increased conductivity and electrocatalytic effect. The increase in the analytical signal can also be attributed to the increase in electroactive surface area promoted by the incorporation of MOF. The electroactive surface areas were estimated using the Randles-Ševčík equation, where measurements were carried out in an aqueous solution containing the [Fe­(CN)_6_)^3‑/4‑^] redox couple. The expression used was

1 $$i_{p}=\left({2.69\times 10}^{5}\right)n^{3/2}{AD}^{1/2}{Cv}^{1/2}$$

where *i*_p_ is the peak current (A), *n* is the number of electrons involved in the redox process, *A* is the electroactive surface area (cm^2^), *D* is the diffusion coefficient (cm^2^ s^–1^), *C* is the concentration of the redox species (mol cm^–3^), and ν is the scan rate (V s^–1^). Using this approach, the calculated electroactive surface areas were (1.35 ± 0.1) mm^2^ for the unmodified electrode and (3.4 ± 0.3) mm^2^ for AuNPs@Al-TDC/3D-CPE.

**Table 1:** Electrochemical Parameter for the Unmodified and MOF-Modified Electrodes

electrode	*I*p_a_/*I*p_c_	area (mm^2^)	ΔEp (mV)	k^0^ (cm s^–1^)	rct (Ω)
3D-CPE	1.020	1.4 ± 0.1	149	2.57 × 10^–3^	5948.4
AuNPs@Al-TDC/3D-CPE	1.094	3.4 ± 0.3	103	5.30 × 10^–3^	5466.4

**Figure 2:**
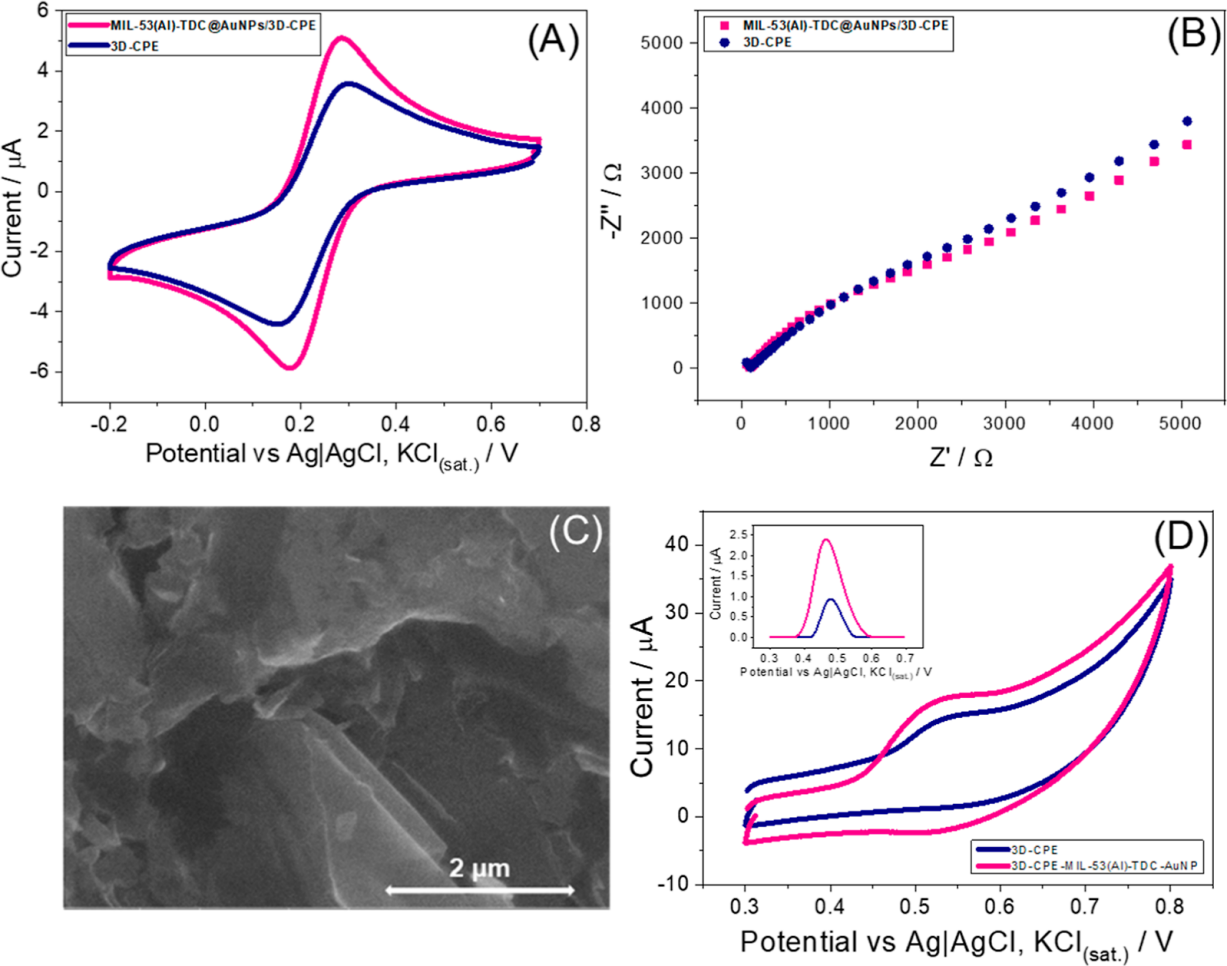
Shows the response for unmodified 3D-CPE (blue line) and the AuNPs@Al-TDC/3D-CPE (pink line) by (A) CV of 5 mmol L^–1^ [Fe­(CN)_6_]^3–^ redox probe. (B) Nyquist plots from EIS of the same redox probe. (C) SEM image of the AuNPs@Al-TDC/3D-CPE sensor surface at 50,000× magnification. (D) Comparison of CV responses for 200 μmol L^–1^ DIU. The inset graph shows DPV voltammograms for 10 μmol L^–1^ DIU, both in 0.1 mol L^–1^ BR buffer solution at pH 12.0. Experimental parameters: CV scan rate, 100 mV s^–1^; DPV amplitude, 80 mV; step potential, 7 mV; modulation time, 50 ms.

A slightly higher reversibility (Δ*E*p) is also observed for the modified electrode (smaller difference between the oxidation and reduction potentials), which suggests faster electron transfer kinetics using the hybrid material, corroborated by the higher value of the heterogeneous electron transfer constant (*k* ^0^) estimated for both electrodes, calculated using the Nicholson eq.^39^ The increase in *k* ^0^ can be attributed to the high conductivity of the AuNPs and the porosity of the MOF, which favors the diffusion of ionic species at the electrochemical interface.

EIS measurements using the same redox couple further revealed differences in charge transfer resistance (Rct) between the electrodes (Figure 2B). The semicircular behavior observed in the curves is typical of processes controlled by charge transfer at the electrode interface. The unmodified 3D-CPE exhibited an Rct of 5948.4 Ω, while the AuNPs@Al-TDC/3D-CPE sensor showed a reduced Rct of 5466.4 Ω. The reduction in Rct after modifying the composite material with MOF and AuNPs shows an improvement in electronic conductivity and ease of electronic transfer. In general, the AuNPs act as a conductive bridge, facilitating charge transfer, while the MOF contributes by increasing the electroactive surface area and improving ion diffusion.

The sensor surface morphology was further characterized by SEM. Images of the unmodified 3D-CPE sensor (Figure S3, Supporting Information) reveal a relatively homogeneous and rough texture, featuring thin layered structures typical of graphite dispersed within the ABS polymer matrix. In contrast, the AuNPs@Al-TDC/3D-CPE sensor (Figure 2C) exhibits noticeably increased surface roughness following modification. The surface morphology is more irregular, with visible clusters of crystalline particles separated by porous spaces. This heterogeneous and rough texture aligns with the characteristic morphology of MOF, whose defining feature is its intrinsic porosity. Elemental composition analysis of Al-TDC, AuNPs@Al-TDC, and AuNPs@Al-TDC/3D-CPE by EDX, presented in Figure S4, confirms the presence of aluminum and gold in the species, verifying the successful incorporation on the electrode.

Finally, the increase in the electroactive area provided by the MOF’s porosity favors greater adsorption of the DIU molecules on the surface of the proposed sensor, which also has homogeneously distributed AuNPs, as shown in Figure S2 of the Supporting Information, and synergistically act as catalytic sites, thus facilitating electron transfer during the analyte’s redox mechanism. This behavior is corroborated by the significant increase in the electrochemical response of DIU, evaluated and compared between the unmodified 3D-CPE and the AuNPs@Al-TDC/3D-CPE sensors using CV (200 μmol L^–1^) and DPV (10 μmol L^–1^), as shown in Figure 2D. In both techniques, the modified sensor exhibited a 2.5-fold increase in the peak current associated with the DIU oxidation process, demonstrating significantly enhanced sensitivity, as well as more efficient electron transfer on the surface of the electrode. However, the peak potential for DIU oxidation showed no substantial displacement, with only a minor variation of approximately 14 mV observed.

### 3.3 Electrochemical Behavior of the DIU on AuNPs@Al-TDC/3D-CPE Surface

The effect of the supporting electrolyte pH (BR buffer 0.1 mol L^–1^) on the redox behavior of DIU (10 μmol L^–1^) was studied by DPV over a pH range of 2.0 to 12.0. Increasing the pH led to a progressive shifting of the anodic peak toward less positive oxidation potentials (Figure 3A), a behavior which indicates that the redox process of DIU involves protons (H^+^). Regarding the peak current, higher values were observed in more acidic conditions, with the current at pH 2.0 nearly three times greater than at pH 12.0 (Figure 3B). Nevertheless, at pH 12.0, the oxidation potential shifted approximately 600 mV toward more cathodic potentials, indicating improved electrochemical favorability. Thus, pH 12.0 was chosen as the optimal condition for the supporting electrolyte.

**Figure 3:**
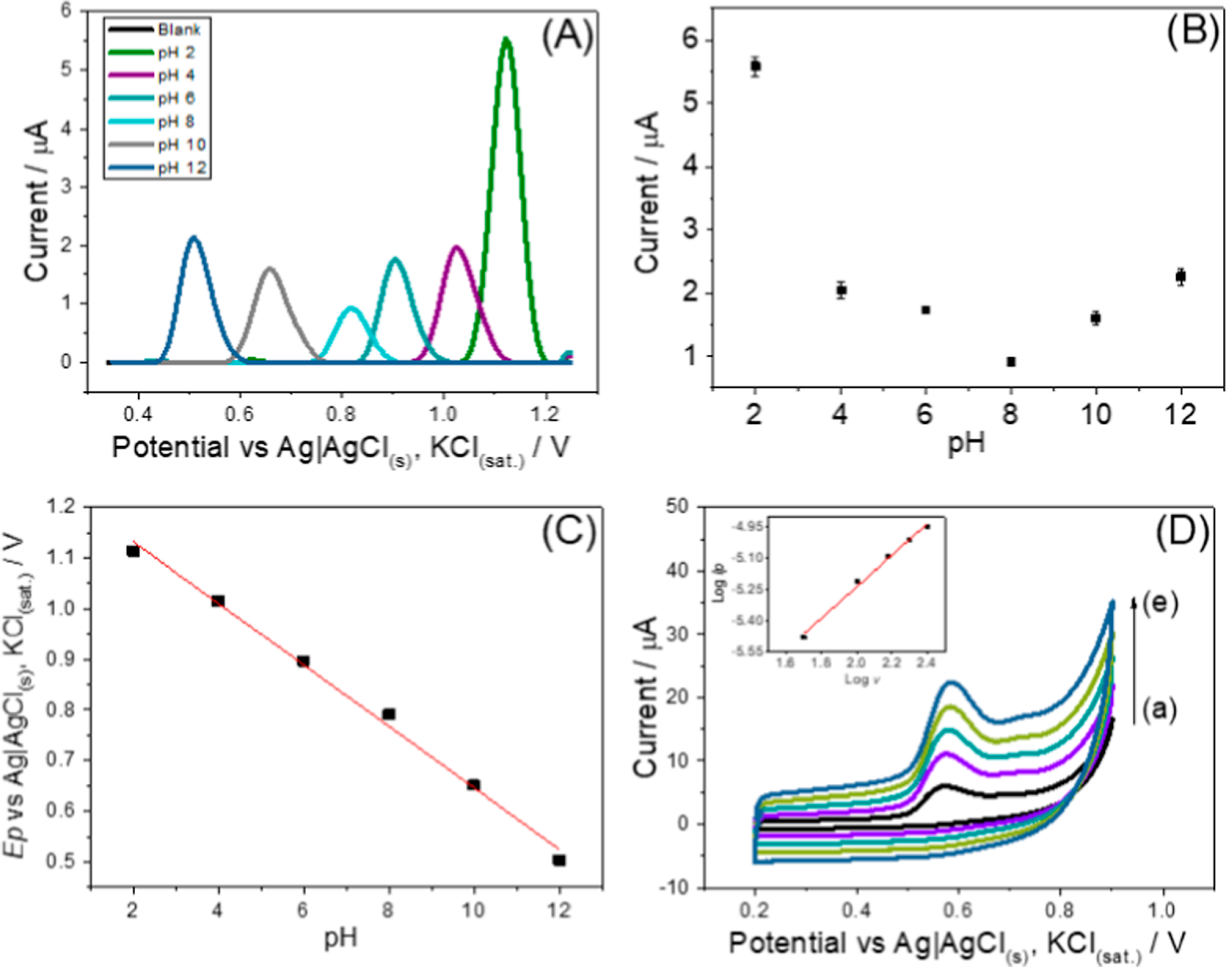
(A) DPV voltammograms of the DIU (10 μmol L^–1^) at different pH values of the supporting electrolyte. Effect of supporting electrolyte pH on the DIU oxidation: (B) peak current and (C) peak potential. (D) CV voltammograms of DIU (250 μmol L^–1^) at scan rates ranging from 50 mV s^–1^ (a) to 250 mV s^–1^ (i). The inserted graph of the logarithmic plot of peak current (Ip) versus scan rate (v). DPV parameters: pulse amplitude 100 mV, step potential 7 mV, modulation time 50 ms. All measurements were performed in BR buffer (0.1 mol L^–1^) as the supporting electrolyte.

The redox behavior of DIU at the AuNPs@Al-TDC/3D-CPE electrode surface exhibited a linear dependence on the pH of the supporting electrolyte (R^2^ = 0.99, Figure 3C), described by the equation: *E*p = (−0.061 ± 0.002) pH + (1.25 ± 0.02). The slope of approximately −61 mV closely matches the theoretical Nernstian value of −59 mV/pH, indicating that the redox process involves an equal number of electrons and protons. Accordingly, the DIU oxidation mechanism likely proceeds through the transfer of one electron and one proton, generating a free radical that subsequently dimerizes, as proposed in Figure S4. This mechanism of DIU oxidation is consistent with previously reported studies.^40,41^

The mass transport behavior of DIU at the modified sensor was investigated by CV, varying the scan rate from 50 to 250 mV s^–1^ in 0.1 mol L^–1^ BR buffer (pH 12.0), as shown in Figure 3D. DIU exhibits an irreversible redox process, with a peak oxidation potential around +0.56 V (vs Ag|AgCl_(s)_, KCl_(sat.)_). A linear relationship was observed between the logarithm of the peak current (Ip) and the logarithm of the scan rate (v) (R^2^ = 0.99), described by the equation: log Ip = (−6.74 ± 0.08) + (0.75 ± 0.04) log *v* (insert in Figure 3D). The slope of approximately 0.75 indicates that the electrochemical process at the AuNPs@Al-TDC/3D-CPE surface is governed by a combination of diffusion and adsorption of DIU on the porous structures of the MOF.^42^ It is suggested that during the potential scan, the DIU molecules are attracted to the electrode surface and adsorb onto the porous structure of the AuNPs@Al-TDC MOF. The AuNPs then act as electrocatalytic sites, facilitating electron transfer and, consequently, the redox reaction, while the oxidation products diffuse back into the solution.

### 3.4 DPV Parameters Optimization

The DPV instrumental parameters for the DIU analysis were systematically optimized. The measurements were conducted using 0.1 mol L^–1^ BR buffer (pH 12.0) as supporting electrolyte and 5 μmol L^–1^ DIU in the electrochemical cell. Amplitude values ranging from 10 to 100 mV (Figures S6A and S6B) and step potentials from 1 to 8 mV (Figures S6C and S6D) were evaluated. Increasing the amplitude led to an enhanced analytical response along with a slight broadening of the peak base. However, this increase in peak current plateaued at around 80 mV, which was therefore selected as the optimal amplitude for subsequent analyses. Similarly, increasing the step potential resulted in higher peak currents and peak broadening. Beyond 6 mV, the improvement in current became less pronounced, and at 8 mV, the voltammogram exhibited a loss of resolution. Consequently, a step potential of 7 mV was deemed optimal, as it balanced improved analytical frequency with minimal loss of resolution compared to 6 mV.

### 3.5 Analytical Curve and Figures of Merit

DPV measurements under the optimized instrumental conditions were used to construct an analytical calibration curve, covering DIU concentrations from 0.99 to 19.2 μmol L^–1^ (Figure 4A). The curve exhibited excellent linearity (*R* ^2^ = 0.999), described by the equation: Ip (μA) = (0.328 ± 0.002) [DIU] (μmol L^–1^) – (0.247 ± 0.004) (Figure 4B). The limits of detection (*L*_D_) and quantification (*L*_Q_) were calculated according to IUPAC guidelines, using the equations *L*_D_ = 3.3 × s_b_/a and *L*_Q_ = 10 × s_b_/a (s_b_ = intercept standard deviation; *a* = calibration curve slope).^50^ The resulting values were 0.040 μmol L^–1^ for *L*_D_ and 0.122 μmol L^–1^ for *L*_Q_, demonstrating the high sensitivity of the developed MOF-based sensor.^51^

**Figure 4:**
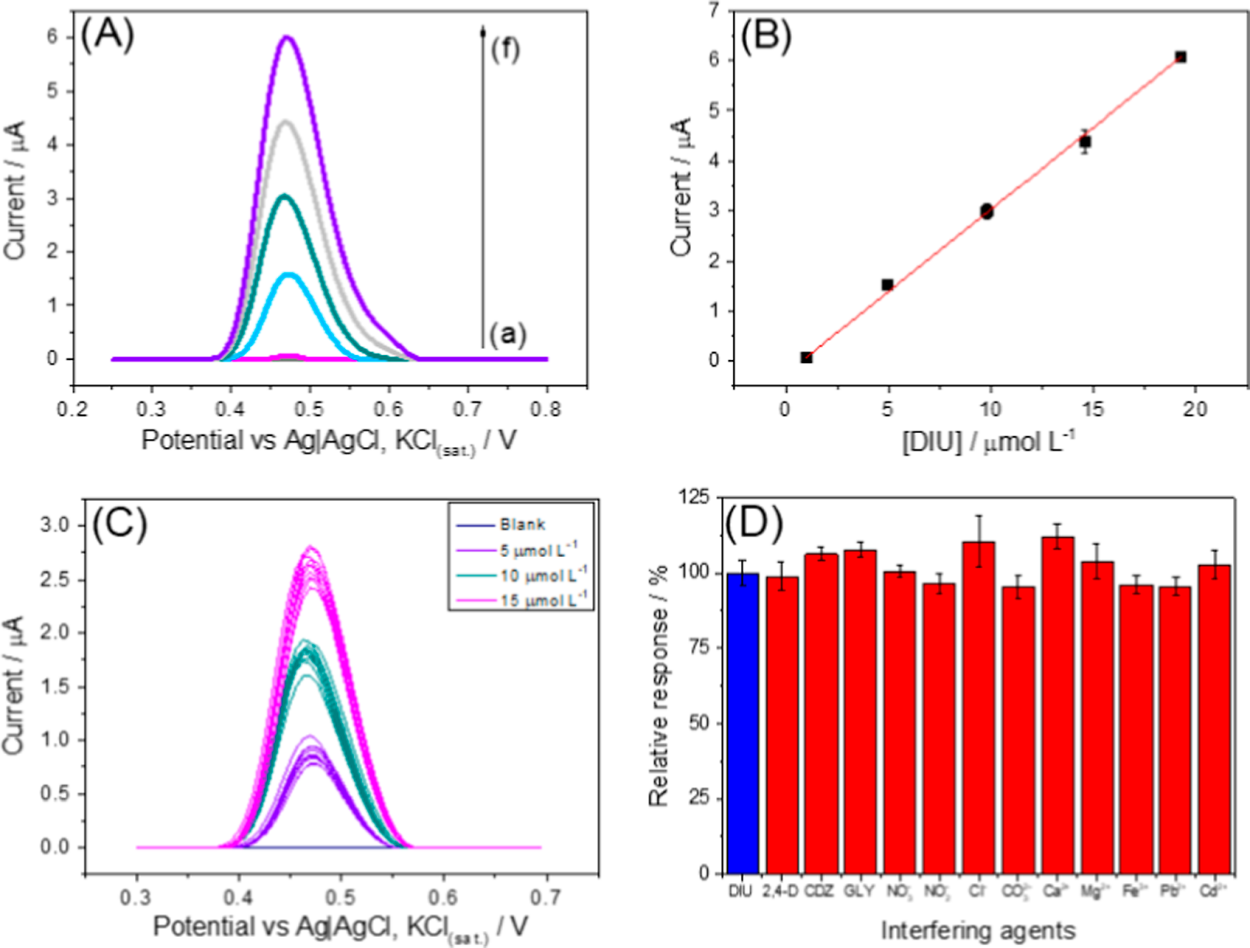
(A) DPV voltammograms for DIU analytical curve in BR buffer 0.1 mol L^–1^ pH 12.0, where (a) blank; (b) 0.99 μmol L^–1^; (c) 4.95 μmol L^–1^; (d) 9.80 μmol L^–1^; (e) 14.56 μmol L^–1^; (f) 19.23 μmol L^–1^. (B) Linear fit of the analytical curve for DIU. (C) Measurements of a solution of DIU (*n* = 10) with concentrations of 5 (purple line), 10 (gray line), and 15 (pink line) μmol L^–1^. (D) Mean values obtained from interference studies for DIU determination (5 μmol L^–1^) for 2,4-*d* (10 μmol L^–1^), CBZ (30 μmol L^–1^), GLY (125 μmol L^–1^), NO_2_^–^ (250 μmol L^–1^), NO_3_^–^ (10 mmol L^–1^), Cl^–^ (7 mmol L^–1^), CO_3_^2–^ (0.3 mmol L^–1^), NO_2_^–^ (250 μmol L^–1^), NO_3_^–^ (10 mmol L^–1^), Ca^2+^ (0.3 mmol L^–1^), Mg^2+^ (0.06 mmol L^–1^), Fe^3+^ (5 μmol L^–1^), Pb^2+^ (5 μmol L^–1^), 0and Cd^2+^ (5 μmol L^–1^). Conditions experimental DPV: amplitude 80 mV; step potential 7 mV; modulation time 50 ms.

A repeatability study was conducted to evaluate the precision of the sensors. Ten consecutive DPV measurements were recorded at three DIU concentrations (5, 10, and 15 μmol L^–1^) in the electrochemical cell, yielding relative standard deviations (RSD) of 10.0%, 8.4%, and 7.1%, respectively (Figure 4C).^52^ These findings confirm that the developed sensor exhibits excellent precision under the optimized DPV conditions for DIU determination.^53^ Furthermore, the analytical reproducibility of the AuNPs@Al-TDC/3D-CPE sensor was evaluated using CV measurements of a potassium ferricyanide redox probe (5 mmol L^–1^) in potassium chloride (1 mol L^–1^).^54^ The RSD obtained for 5 independent devices was 1.6%, showing the high reproducibility of the manufacturing process of the proposed sensor (Figure S7 of the Supporting Information).

The stability of the AuNPs@Al-TDC/3D-CPE sensor was evaluated over 24 days. The analytical response of the sensor remained stable during this time with a variation in the average response between days 1 (RSD = 3.8%) and 24 (RSD = 8.7%) of 2.1%, for a 5 μmol L^–1^ solution of DIU in BR buffer pH 12.0, as shown by Figure S8A. Stability throughout the measurement process was also verified through 50 CV cycles using the 5 mmol L^–1^ potassium ferricyanide redox probe in a 1 mol L^–1^ potassium chloride medium, as shown in Figure S8B, with minimal variation between the first and fiftieth cycles, which indicates good stability of the proposed sensor. On the other hand, the selectivity of the sensor was assessed through interference studies during the voltammetric determination of DIU by DPV. Potential interferents such as Cl^–^ (7 mmol L^–1^), CO_3_^2–^ (0.3 mmol L^–1^), NO_2_^–^ (250 μmol L^–1^), NO_3_^–^ (10 mmol L^–1^), Ca^2+^ (0.3 mmol L^–1^), Mg^2+^ (0.06 mmol L^–1^) and Fe^3+^ (5 μmol L^–1^) were evaluated at concentrations common in natural waters, whereas some widely used pesticides such as 2,4-*d* (10 μmol L^–1^), CBZ (30 μmol L^–1^) and GLY (125 μmol L^–1^), were studied at concentrations corresponding to their Maximum Residual Limit (MRL) values in water samples. On the other hand, heavy metals such as Pb^2+^ (5 μmol L^–1^) and Cd^2+^ (5 μmol L^–1^) were evaluated at concentrations (at least 500 times) considerably higher than those permitted in natural waters. In all experiments, the DIU concentration was maintained at 5 μmol L^–1^ in 0.1 mol L^–1^ BR buffer solution (pH 12.0). The DIU response exhibited variations of no more than 12% (Figures 4D and S9), confirming that the AuNPs@Al-TDC/3D-CPE sensor provides reliable selectivity for DIU determination, even in the presence of common agricultural pesticides and typical components of tap, lake, and river water samples.

### 3.6 Sample Analysis

High environmental stability and persistence coupled with excessive use of DIU, has led to its frequent detection in aqueous matrices.^43^ Reported concentrations in surface water samples range from 0.05 to 0.63 μmol L^–1^,^1,44–46^ highlighting the demand for sensitive and selective analytical methods for their monitoring. In this context, the AuNPs@Al-TDC/3D-CPE sensor was employed to determine DIU levels in real water samples, including tap, river, and lake sources. DIU concentrations in all samples were below the L_D_. Consequently, recovery tests were performed in order to evaluate the accuracy of the method by spiking the samples at three concentration levels: 5.0, 10.0, and 15.0 μmol L^–1^.

The measured concentrations in each spiked sample are summarized in Table 2, together with the corresponding recovery percentages, which ranged from 97% to 105%. These results highlight the high accuracy of the proposed AuNPs@Al-TDC/3D-CPE sensor for DIU determination in water samples. Furthermore, the sample preparation was straightforward and rapid, involving just two steps: filtration through paper and a 10-fold dilution in the supporting electrolyte.

**Table 2:** Average Recovery Percentages (*n* = 3) for DIU in Spiked Tap, River, and Lake Water Samples Determined by DPV Using the AuNPs@Al-TDC/3D-CPE Sensor

samples	added (μmol L^–1^)	found (μmol L^–1^)	recovery (%)
tap water	0	*L*_D_	-
	5.0	4.9 ± 0.3	99 ± 6
	10.0	9.9 ± 0.5	99 ± 5
	15.0	15.7 ± 0.6	105 ± 4
river water	0	*L*_D_	-
	5.0	4.9 ± 0.4	98 ± 7
	10.0	10.4 ± 0.5	104 ± 5
	15.0	15.6 ± 0.9	104 ± 6
lake water	0	*L*_D_	-
	5.0	5.1 ± 0.3	102 ± 5
	10.0	9.7 ± 0.7	97 ± 7
	15.0	15.6 ± 1.2	104 ± 8

### 3.7 Comparison of the Electrochemical Performance of the AuNPs@Al-TDC/3D-CPE Sensor

The main figures of merit of the method developed in this study were compared with those of other electrochemical sensors previously reported for DIU determination, as summarized in Table 3. The AuNPs@Al-TDC/3D-CPE sensor stands out for its relatively wide linear range, comparable to that of other sensors, while maintaining a *L*_D_ in the same order of magnitude. In general, most sensors reported in the literature, including the one developed in this work, have been applied to the analysis of water samples. Some sensors, such as rGO/GCE, have also been employed for more complex matrices like tap water, grape juice, and orange juice, but exhibited significantly higher *L*_D_ compared to the disposable sensor proposed here.^47^ Although the CuO/WON/GCE sensor achieved a lower L_D_ for both water and juice samples,^48^ it is worth noting that it involved a commercially available electrode with multiple surface modifications, unlike the low-cost, disposable platform developed in this study.^49^

**Table 3:** Comparative Analysis of the Analytical Performance of the AuNPs@Al-TDC/3D-CPE Sensor with Other Electrochemical Sensors Previously Reported for DIU Determination^a^

electrode	technique	linear working range (μmol L^–1^)	*L*_D_ (μmol L^–1^)	samples	reference
**CuO/WON/GCE**	DPV	0.01–764.4	0.006	tap and river water, grape and orange juice	^49^
**MWCNT-COOH-MIP/CPE**	SWV	0.05–1.25	0.009	river water	^8^
**CMO NPs/f-BN/GCE**	DPV	0.01–1770	0.01	river and lake water	^50^
**BDD**	DPV	1–9	0.04	lake and groundwater	^51^
**3D-CPE**	SWV	0.25–20	0.07	sugar cane juice and cachaça	^52^
**PtNPs/CS/GCE**	DPVAdSV	0.17–4.29	0.09	river water	^53^
**rGO/GCE**	BIA-AMP	5–50	0.40	tap water, grape, and orange water	^48^
**rGO-AuNPs/SPE**	LSV	2.15–128.7	0.54	lake and seawater	^53^
**(p-Phe)-PGE**	DPV	10–50	43.4	water	^54^
**AuNPs@Al-TDC/3D-CPE**	DPV	0.99–19.2	0.04	tap, river, and lake water	this work

a **CuO/WON/GCE**: glassy carbon electrode modified with oxynitride oxide and copper oxide; **MWCNT-COOH-MIP/CPE**: carbon paste electrode modified with a molecularly imprinted polymer and carboxyl-functionalized multiwalled carbon nanotubes; **CMO NPs/f-BN/GCE**: glassy carbon electrode modified with nanocomposite of mixed spinel metal oxide [Co, Mn oxides] and functionalized boron nitride; **BDD**: boron-doped diamond electrode; **PtNPs/CS/GCE**: glassy carbon electrode modified with platinum nanoparticles and chitosan; **rGO/GCE**: glassy carbon electrode modified with reduced graphene oxide; **rGO-AuNPs/SPE**: screen-printed electrode modified with reduced graphene oxide and gold nanoparticles; **(p-Phe)-PGE**: pencil graphite electrode modified by electropolymerization of p-phenylenediamine; **SWV**: square wave voltammetry; **DPVAdSV**: differential pulse adsorptive stripping voltammetry; **BIA-AMP**: batch injection analysis with amperometric detection; LSV: linear sweep voltammetry.

Conversely, low-cost sensors, including carbon paste electrodes (CPE), screen-printed electrodes (SPE), and pencil graphite electrodes (PGE), have also been utilized for DIU detection Among these, the MWCNT–COOH–MIP/CPE sensor^9^ achieved the best detectability, exhibiting the lowest detectability. However, despite its sensitivity, this sensor involves a complex and costly fabrication process due to surface modifications and the use of molecularly imprinted polymers, in addition to presenting a relatively narrow linear range.

In this context, the AuNPs@Al-TDC/3D-CPE electrode stands out for its excellent analytical performance for DIU determination, while also offering low production cost and incorporating recycled materials. These features position the proposed sensor as a promising and feasible alternative for routine analytical applications. Furthermore, comparison with previously reported sensors demonstrates that it is now possible to design cost-effective and innovative strategies that adhere sustainability principles without compromising analytical efficiency and, in some cases, even surpassing conventional approaches.

## 4 Conclusions

This study reports on the development of a novel composite material modified with an MOF for the construction of a 3D printed sensor. The sensor was fabricated using recycled ABS waste from 3D printing, powdered graphite, and Al-TDC MOF modified with AuNPs. The fabrication process was simple, rapid, and cost-effective, enabling the reuse of polymeric waste and contributing significantly to the sustainability of the proposed analytical method. The work is aligned with the United Nations Sustainable Development Goals (SDGs) 6, 9 and 12, which address drinking water, innovation and responsible consumption and production, respectively.

The MOF material and the AuNPs@Al-TDC/3D-CPE sensor, were thoroughly characterized using spectroscopic, imaging, and voltammetric techniques, which confirmed the enhanced performance of the modified sensor compared to its unmodified counterpart. Additionally, the electrochemical performance of the proposed sensor was benchmarked against other devices reported in the literature, demonstrating comparable or superior analytical figures of merit. Main advantages include a wide dynamic linear range, excellent detectability in the nanomolar range, and satisfactory precision and accuracy.

The synergistic combination of recycled polymer-based composites with MOFs imparts promising electroanalytical and sustainable properties, enabling the fabrication of inexpensive sensors with outstanding performance. Moreover, the results indicate significant potential for the development of novel materials, such as MOF-modified conductive filaments for 3D printing, broadening the application scope of MOF-based 3D-printed sensors toward the detection of diverse emerging contaminants.

## Supplementary Material



## Notes

Electrochemical sensor construction process images; SEM images; EDS spectra and elemental composition; DIU electrochemical redox mechanism; instrumental optimization voltammograms; reproducibility, stability and selectivity tests (PDF)
