# Supplementary material for: AuNPs@Al-TDC Metal–Organic Framework: A Hybrid Nanostructure for 3D-Printed Electrochemical Sensors Targeting Diuron
Source: ACS Omega. 2026 Feb 11;11(7):12242–53. doi: 10.1021/acsomega.5c11545 (PMC12947146; doi:10.1021/acsomega.5c11545)
Supplement: Supplementary file 1 [file ao5c11545_si_001.pdf]

Supplementary Material

AuNPs@Al-TDC Metal–Organic Framework: A Hybrid Nanostructure for 3D-Printed Electrochemical Sensors Targeting Diuron

Raylla Santos Oliveira[a], Hudson Batista da Silva[a], Esther de Jorge Duarte[a], Cassiano Cunha de Souza[a], Wallace Burger Veríssimo de Oliveira[a], Charlane Cimini Corrêa[a], Gustavo Fernandes Souza Andrade[a], Maria Auxiliadora Costa Matos[a], Thalles Pedrosa Lisboa[b], and Renato Camargo Matos[a]*

[a]*Departamento de Química, Universidade Federal de Juiz de Fora, 36026-900, Juiz de Fora-MG, Brazil*

[b]*Faculdade de Ciências Exatas e Tecnologia, Universidade Federal da Grande Dourados, 79804-970, Dourados-MS, Brazil*

*Corresponding author: *renato.matos@ufjf.br*

Phone number: +5532988310321

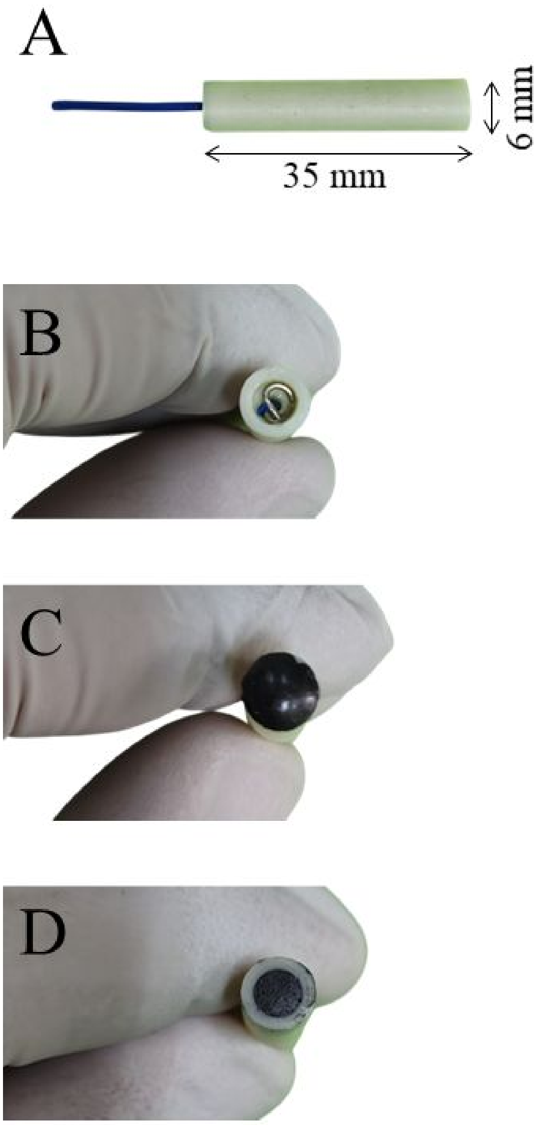

Figure S1. Real images of the manufacturing process of the AuNPs@Al-TDC/3D-CPE sensor showing: **(A)** lateral view and **(B)** top view of the 3D-printed support; **(C)** 3D-printed support filled with composite material; **(D)** AuNPs@Al-TDC/3D-CPE sensor after the surface polishing step with sandpaper.

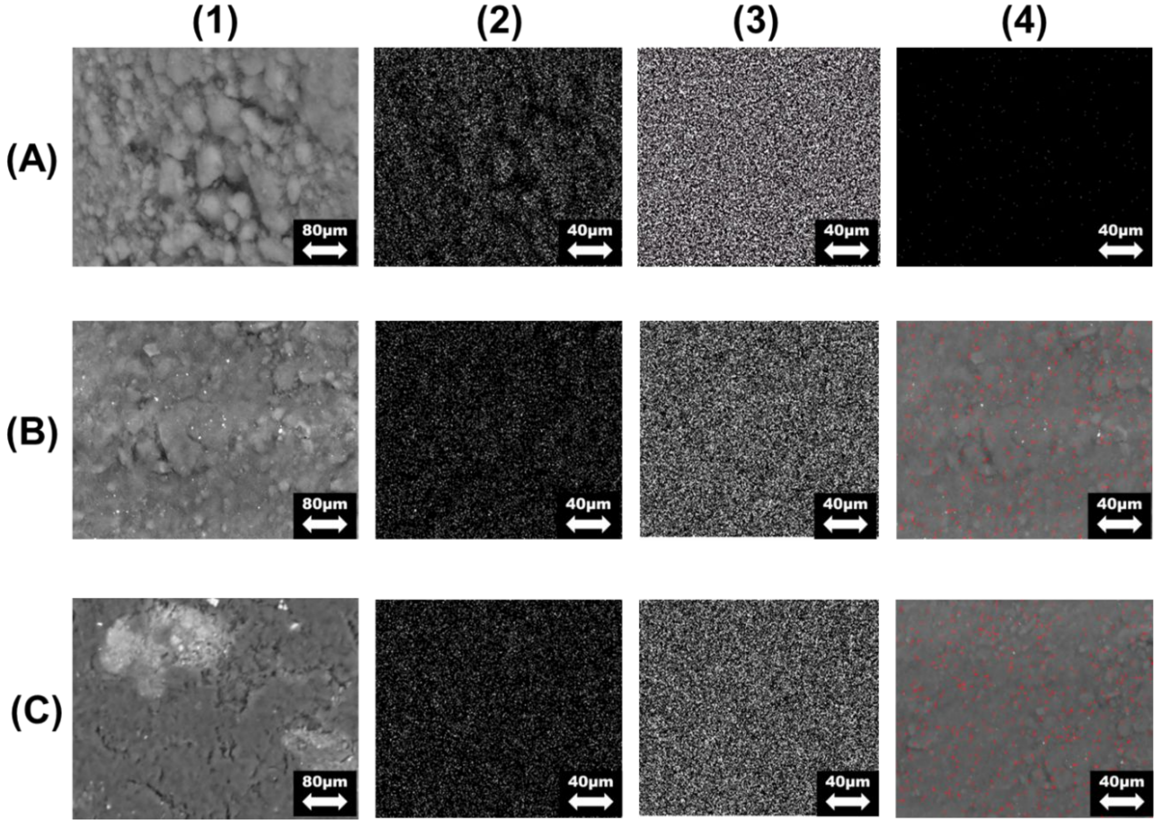

Figure S2. **(1)** SEM images and mapping for **(2)** Carbon, **(3)** Aluminum and **(4)** Gold for **(A)** Al-TDC, **(B)** AuNPs@Al-TDC, and **(C)** AuNPs@Al-TDC/3D-CPE, respectively.

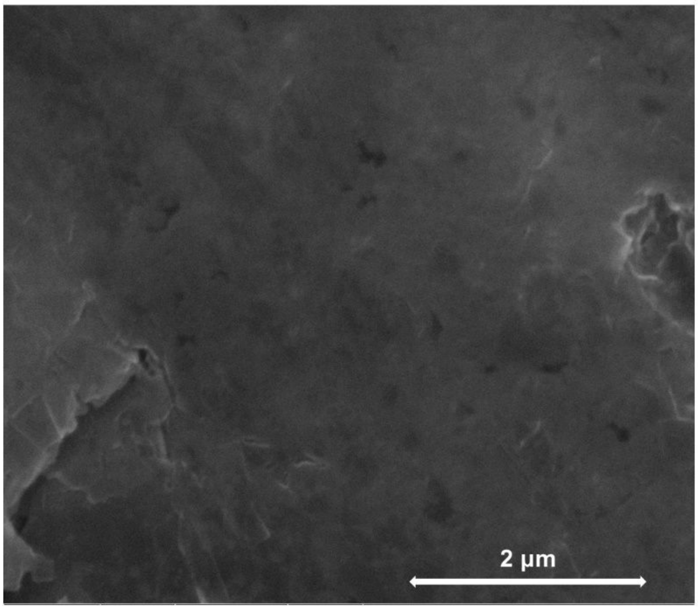

Figure S3. SEM image of the cleaned sensor surface at 50000x magnification.

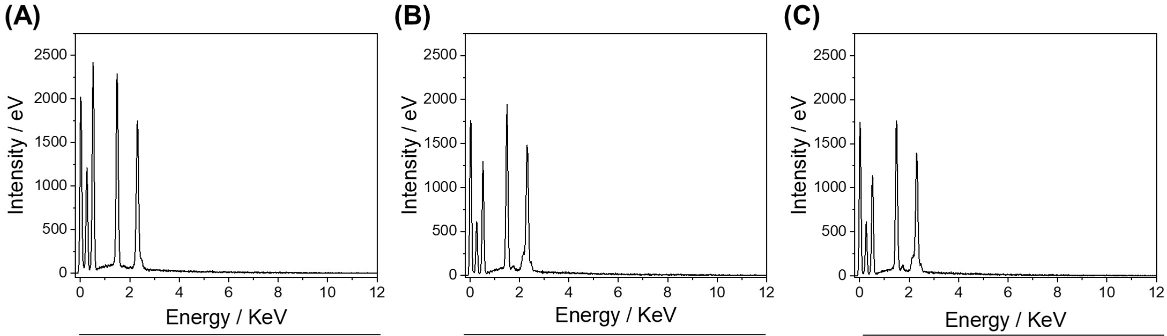

(A)

Element	Atomic %	Weight %
Carbon	47.6	37.6
Oxygen	44.0	46.3
Aluminum	4.4	7.9
Sulfur	3.8	8.1
Gold	0.0	0.0

(B)

Element	Atomic %	Weight %
Carbon	48.7	36.1
Oxygen	39.4	39.0
Aluminum	5.8	9.7
Sulfur	5.6	11.2
Gold	0.3	4.0

(C)

Element	Atomic %	Weight %
Carbon	48.8	36.2
Oxygen	39.1	38.6
Aluminum	5.8	9.8
Sulfur	5.7	11.3
Gold	0.3	4.0

Figure S4. EDS spectrum and elemental composition of the **(A)** Al-TDC, **(B)** AuNPs@Al-TDC, and **(C)** AuNPs@Al-TDC/3D-CPE, respectively.

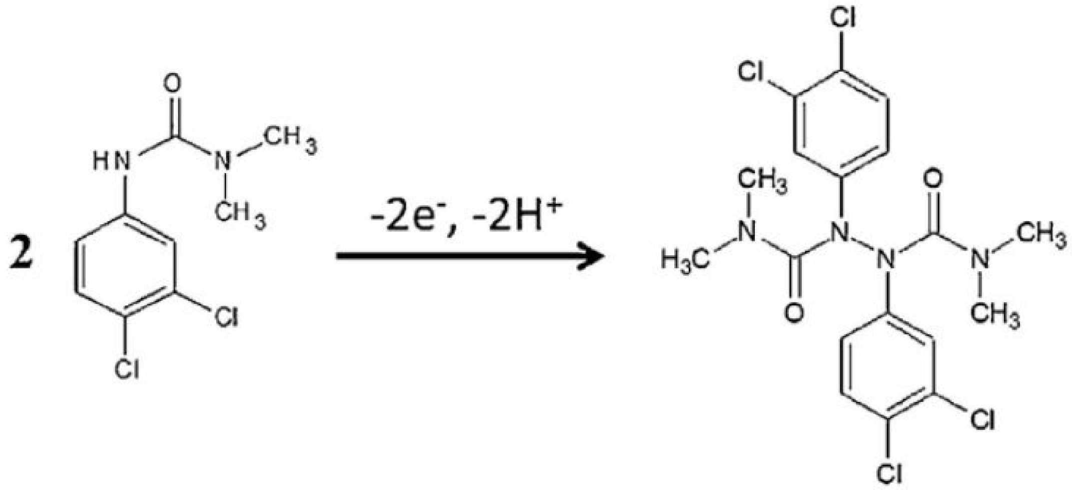

Figure S5. Electrochemical mechanism for the oxidation process of the DIU on the sensor surface.

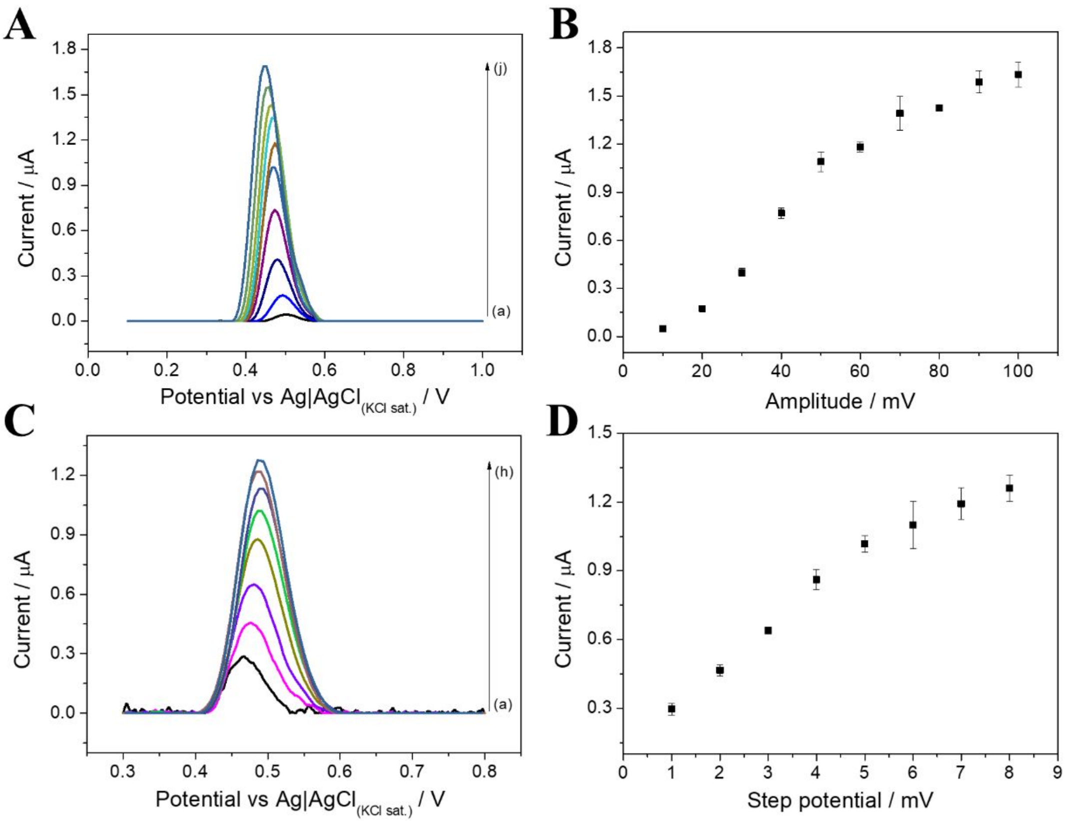

Figure S6. Optimization of DPV instrumental conditions. (A) Amplitude voltammograms (ranging from (a) 10 to (j) 100 mV), (B) amplitude x current graph, (C) potential step voltammograms (ranging from (a) 1 to (h) 8 mV), (D) step and potential x current graph. 5 µmol L^{-1} of DIU in 0.1 mol L^{-1} BR buffer solution, pH 12.0.

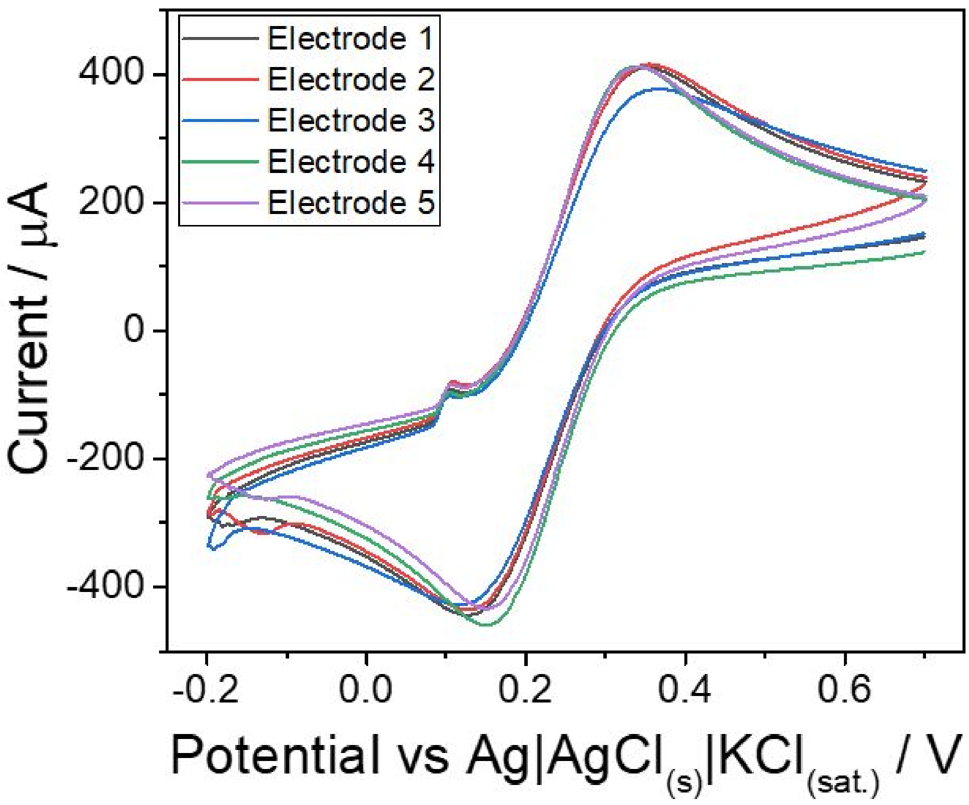

Figure S7. CV measurements of a 5 mmol L^{-1} potassium ferricyanide redox probe in 1 mol L^{-1} potassium chloride for five independently constructed electrodes.

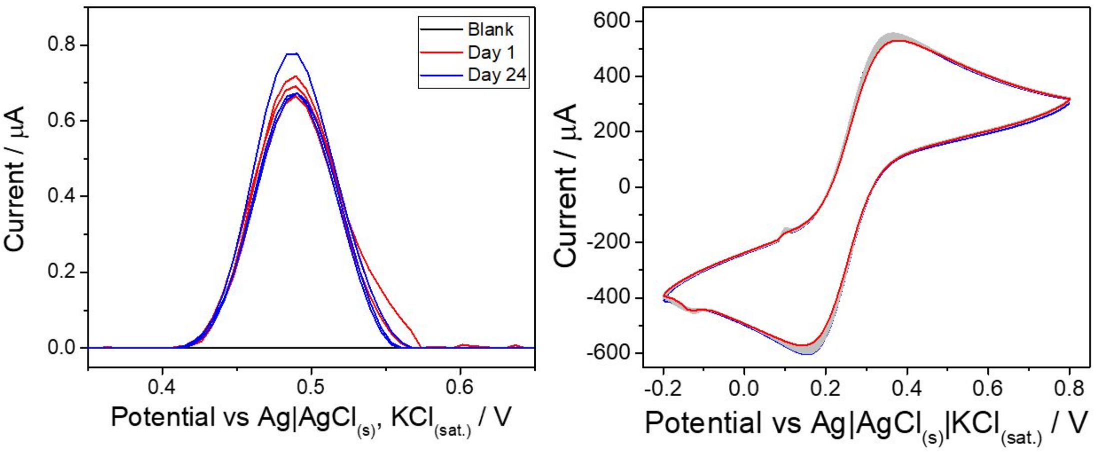

Figure S8. Stability test for the AuNPs@Al-TDC/3D-CPE sensor (A) over 24 days by DPV using a 5 µmol L^{-1} solution of DIU in BR buffer pH 12.0; (B) (B) over 50 cycles by CV using a 5 mmol L^{-1} potassium ferricyanide redox probe in KCl 1 mol L^{-1} where the first cycle is highlighted in blue line and the fiftieth cycle is highlighted in red line.

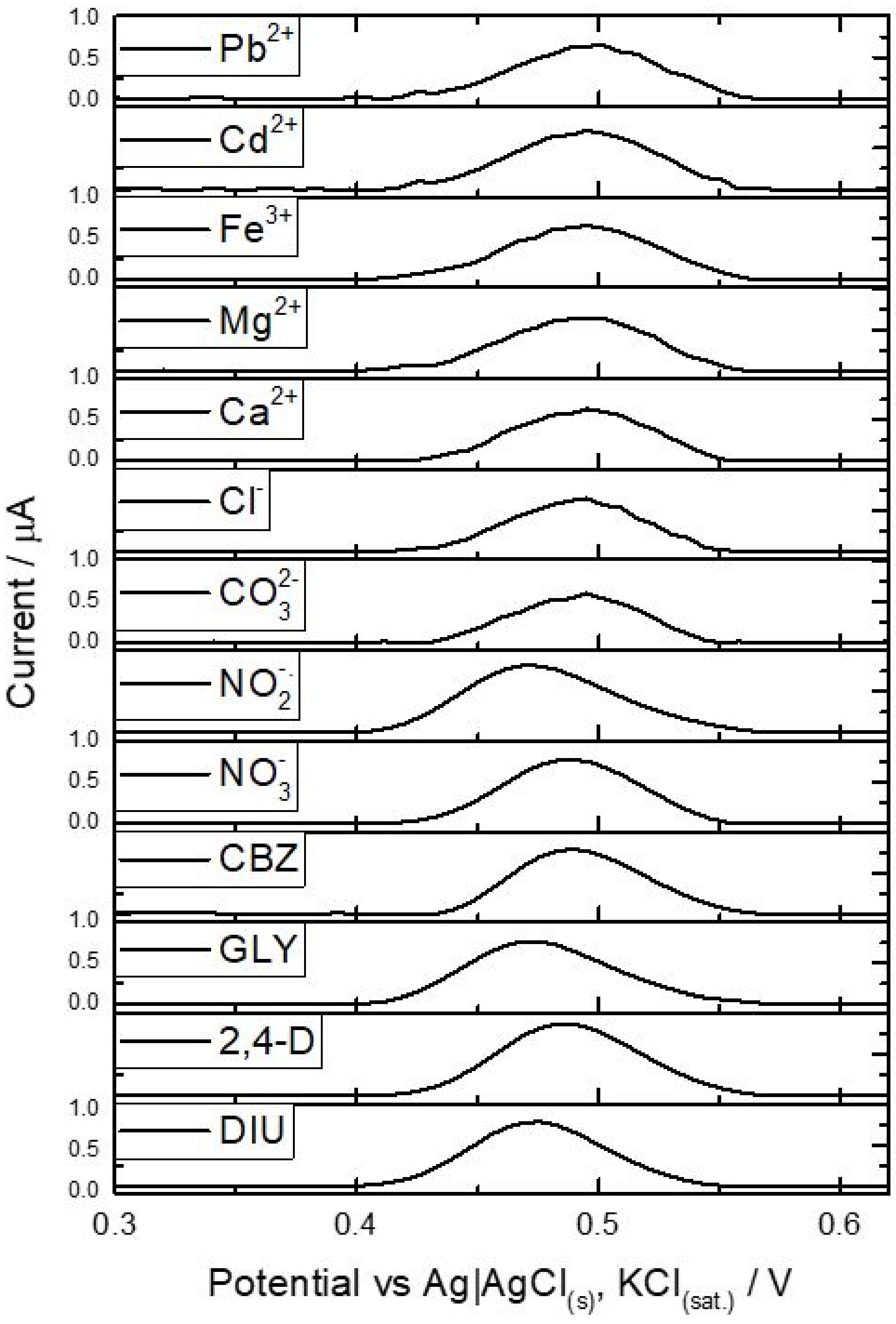

Figure S9. DPV voltammograms for interference test measurements using DIU 5 µmol L^{-1} in 0.1 mol L^{-1} BR buffer solution (pH 12.0) and 2,4-D (10 µmol L^{-1}), CBZ (30 µmol L^{-1}), GLY (125 µmol L^{-1}), Cl^- (7 mmol L^{-1}), CO_3^{2-} (0.3 mmol L^{-1}), NO_2^- (250 µmol L^{-1}), NO_3^- (10 mmol L^{-1}), Ca^{2+} (0.3 mmol L^{-1}), Mg^{2+} (0.06 mmol L^{-1}), Fe^{3+} (5 µmol L^{-1}), Pb^{2+} (5 µmol L^{-1}), and Cd^{2+} (5 µmol L^{-1}).
